# Physical Therapy Intervention With Hippotherapy (HPOT) Affects Balance Control in the Elderly: A Randomized Controlled Trial

**DOI:** 10.1002/hsr2.72349

**Published:** 2026-05-03

**Authors:** Bita Bahrami Gholami, Ali Fatahi, Neda Boroushak, Rozhin Molavian

**Affiliations:** ^1^ Department of Physical Education and Sports Sciences CT.C, Islamic Azad University Tehran Iran; ^2^ Department of sports biomechanics Sport Science Research Institute Tehran Iran

**Keywords:** balance, biomechanics, elderly, falling, hippotherapy, horse riding

## Abstract

**Background and Aims:**

Aging is characterized by a decline in functional abilities, strength, balance, flexibility, agility, and coordination due to neurological and muscular changes. Hippotherapy (HPOT) has been recognized for its physical and psychological benefits for older adults. Hence, this study aimed to investigate the effects of hippotherapy on balance in elderly individuals. The novelty lies in using the Biodex Balance System SD for dynamic balance assessment in healthy elderly without neurological disorders.

**Methods:**

We recruited elderly men aged 60 to 70 who had independent mobility and medical clearance for hippotherapy, ensuring they met criteria such as consistent participation and the absence of pertinent diseases, orthopedic issues, recent surgeries, or medications that could influence outcomes. The dependent variables measured with the Biodex Balance System SD included (I) fall risk, (II) stability index, (III) balance index, (IV) anterior‐posterior deviation, and (V) mediolateral deviation. The intervention consisted of 16 sessions of hippotherapy over 8 weeks, with two 30‐min sessions each week, during which participants rode a horse.

**Result:**

The main findings derived from this study indicate that the intervention significantly reduced Fall Risk in the training group compared to the control (*p* = 0.000, η = 0.77). Hippotherapy also significantly improved Stability index (*p* = 0.041, η = 0.15), Anterior‐Posterior Sway (*p* = 0.013, η = 0.39), mediolateral sway (*p* = 0.002, η = 0.54), and Balance index (*p* = 0.008, η = 0.43). This suggests a noteworthy distinction across all measured variables.

**Conclusions:**

The findings demonstrate that hippotherapy significantly enhances balance in physically active elderly individuals, potentially improving their quality of life. These results will inform professionals working with this population.

## 1 Introduction

Achieving balance in the lives of elderly individuals is crucial for fostering their well‐being and enhancing their quality of life [1]. As people age, they often face various physical, emotional, and social challenges that require a nuanced and multifaceted approach to care [2]. Addressing these challenges is essential not only for the elderly individuals themselves but also for their families and the broader community. Their daily activities may be restricted due to the fear of falls resulting in functional loss, which may further increase the risk of falls. As a result, activities gradually decrease, leading to a deterioration in postural balance of elderly persons [3]. Therefore, balance training can improve elderly persons' stability and reduce the risk of falls [4].

As a result of the phenomenon of population ageing, there are ongoing discussions regarding the functional capabilities of the elderly, as the likelihood of diseases, disabilities, and reliance on healthcare increases with age [5, 6]. Empirical evidence consistently documented in the literature indicates that advancing age correlates with physiological alterations that lead to a decline in muscular strength. Metrics of muscular function, including strength and balance, serve as critical determinants for the preservation and enhancement of functional abilities; furthermore, these same metrics have the potential to forecast the risk of chronic disabilities and diseases [7]. One approach to avert or ameliorate such limitations is to provide guidance aimed at achieving improved functional mobility through the implementation of physical exercises [8]. An intervention deemed protective to alleviate the adverse effects of ageing is the engagement in regular exercise.

Hippotherapy (HPOT) is a physical, occupational, and speech‐language therapy treatment strategy that utilizes equine movement as part of an integrated intervention program to achieve functional outcomes (American Hippotherapy Association, 2024). This approach employs the rhythmic and three‐dimensional movements of equines to elicit neuromuscular reactions through the activation of postural reflex mechanisms in the rider [9]. Given that this form of physical engagement necessitates the utilization of the entire body, the resulting motions can be harnessed to enhance muscular strength, balance, and coordination [4]. Overall, the predominant published evidence supporting the therapeutic effectiveness of hippotherapy has been established within the domain of neurological rehabilitation for disorders such as multiple sclerosis [10], autism [11], Down syndrome [12], and particularly in cases of cerebral palsy [13, 14, 15].

Nevertheless, the implications of hippotherapy for geriatric populations warrant further investigation. Hippotherapy is characterized as a therapeutic strategy that incorporates the movements of horses as an integral component of a comprehensive intervention framework aimed at achieving functional outcomes [16, 17]. As this mode of physical activity involves the engagement of the entire body, the associated movements can be utilized to foster muscle strength, balance, and coordination [4].

It is hypothesized that hippotherapy can enhance the balance and therefore the functions of elderly people and prevent falls, though falls were not directly assessed. To our knowledge, the only controlled clinical trial we assessed about the effect of hippotherapy in elderly showed significant improvement in functional mobility measured after 16 sessions of hippotherapy in healthy and independent individuals [3]. Therefore, the current study aimed to examine the effects of a hippotherapy training intervention on balance variables in elderly people, to determine if this is an effective intervention for this population.

## 2 Methods

This study consisted of a randomized controlled trial with a convenience sampling method.

### 2.1 Sample

We recruited elderly men aged 60 to 70 years with independent mobility and medical clearance for hippotherapy, based on the following criteria: residents of Tehran, ability to participate regularly (attending at least 90% of sessions), no history of diseases affecting dependent variables (e.g., Parkinson's disease, stroke, or vestibular disorders), no orthopedic issues, no recent surgeries, and no medication that could influence results. Elderly participants with osteoporosis diagnosis or obesity (BMI > 30 kg/m²) were excluded. This study received approval from the Research Ethics Committee (REC) at the Kinetic Sciences Research Institute under number IR‐KHU.KRC.1000.193. All participants provided written informed consent.

**Figure 1:**
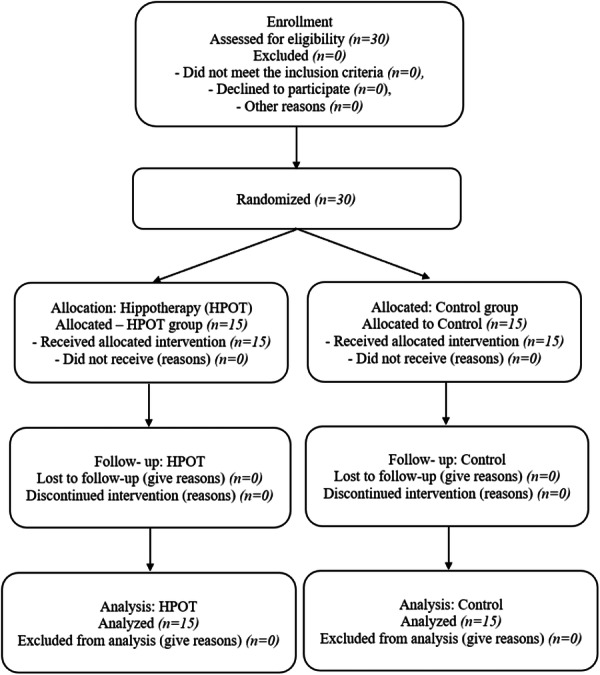
CONSORT 2025 flow diagram of participant progress through the trial.

Participant flow: Participant flow: 30 participants were recruited and randomized (15 to the hippotherapy group and 15 to the control group); all participants completed the study with no losses. All randomized participants (*n* = 30) were included in the primary and secondary outcome analyses in the groups to which they were originally randomized. Participant flow through the trial is shown in the CONSORT 2025 flow diagram (Figure 1). The sample's description characteristics are shown in Table 1. At baseline, there were no discernible differences between the groups, and all dependent variables showed normalcy. Tables 2 and 3 present descriptive and inferential statistics of the dependent variables, respectively. Participants were randomized using a simple random allocation method via a computer‐generated random number table, with allocation concealed in sealed opaque envelopes. Screening involved medical history review and physician clearance. The control group was selected from the same population and received no intervention, continuing their usual activities. Assessors were blinded to group allocation.

**Table 1:** Descriptive characteristics of the study participants.

Groups	Age (years)		Height (cm)		Weight (kg)		BMI (kg/cm^2^)	
	Mean	SD	Mean	SD	Mean	SD	Mean	SD
Experimental group (*n* = 15)	68.6	2.73	1.72	0.04	76.54	8.76	23.12	2.90
Control group (*n* = 15)	69.1	2.03	1.75	0.04	72.25	6.53	22.63	1.76
Significance	0.68		0.42		0.10		0.13	

**Table 2:** Descriptive statistics of dependent variables (means and standard deviations) pre‐ and post‐test.

Variables	Groups	Pre test		Post test	
		Mean	Std. deviation	Mean	Std. deviation
Fall risk	Control	7.14	2.69	7.35	2.36
	Experimental	5.80	3.19	2.12	0.70
	Total	6.49	2.93	4.73	3.18
Stability index	Control	1.16	0.97	1.56	1.44
	Experimental	0.47	0.37	0.26	0.08
	Total	0.82	0.79	0.91	1.20
Anterior posterior sway (cm)	Control	0.68	0.58	0.49	0.36
	Experimental	0.59	0.58	0.20	0.09
	Total	0.64	0.56	0.34	0.30
Mediolateral sway (cm)	Control	0.92	1.49	0.51	0.30
	Experimental	0.81	1.53	0.18	0.05
	Total	0.86	1.46	0.34	0.27
Balance index	Control	1.39	0.38	1.44	0.48
	Experimental	1.25	0.19	0.76	0.35
	Total	1.31	0.29	1.10	0.54

**Table 3:** Inferential analyses of the dependent variables.

Tests of between‐ participants effects							
Dependent variable	Source	Type III sum of squares	*df*	Mean square	F	Sig.	Partial Eta squared
FR	Corrected model	126.22^a^	2	63.11	31.99	0.000	0.83
	Intercept	13.40	1	13.40	6.79	0.022	0.343
	FR pre	16.86	1	16.86	8.54	0.012*	0.40
	Groups	84.56	1	84.56	42.87	0.000*	0.77
SI	Corrected model	14.77^a^	2	7.39	14.42	0.001	0.69
	Intercept	0.03	1	0.03	0.06	0.806	0.01
	SI pre	7.93	1	7.93	15.48	0.002*	0.54
	Groups	1.14	1	1.14	2.22	0.041*	0.15
AP	Corrected model	0.88^a^	2	0.44	12.46	0.001	0.66
	Intercept	0.12	1	0.12	3.31	0.092	0.20
	AP pre	0.53	1	0.53	14.90	0.002*	0.53
	Groups	0.29	1	0.29	8.18	0.013*	0.39
ML	Corrected model	0.74^a^	2	0.37	14.18	0.001	0.69
	Intercept	0.77	1	0.77	29.73	0.000	0.70
	ML pre	0.31	1	0.31	11.81	0.004*	0.48
	Groups	0.40	1	0.40	15.46	0.002*	0.54
BI	Corrected model	2.58^a^	2	1.29	9.62	0.003	0.60
	Intercept	0.01	1	0.01	0.07	0.796	0.01
	BI pre	0.71	1	0.71	5.32	0.038*	0.29
	Groups	1.29	1	1.29	9.63	0.008*	0.43

Abbreviations: AP, anterior‐posterior sway; BI, balance index; FR, fall risk; ML, mediolateral sway; SI, stability index.

^a^ R Squared = 0.831 (Adjusted R Squared = 0.805).

* Significant differences (*p* < 0.05).

### 2.2 Instruments

**Figure 2:**
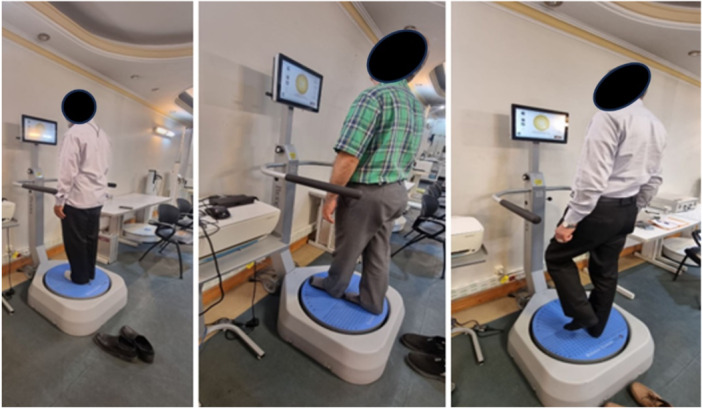
Biodex® Balance System SD platform used for balance assessments.

Data were collected in the clinical laboratory at Islamic Azad University, Tehran. Dependent variables of the study were considered: (I) Fall risk, (II) Stability index, (III) Balance index, (IV) Anterior posterior deviation, (V) Mediolateral deviation. All variables measured by the Biodex Balance System SD (Biodex Medical Systems, Shirley, NY, USA). The participant stands on an adjustable platform equipped with precise sensors. This platform can be modified to simulate various instabilities. Depending on the type and purpose of the test, the balance platform may be positioned differently (fixed or movable), or the participant may be required to stand on it with eyes either open or closed [18]. For fall risk and stability tests, the platform was movable with eyes open; for balance index, it was fixed with eyes closed. In this test, the participant stands on a Biodex platform and maintains balance in a perfectly static position. The test typically lasts 20 s and is repeated under different conditions (eyes open and closed, stable and unstable surface) (Figure 2).

### 2.3 Procedures

Pre‐test functional evaluation tests were administered the week prior to the intervention and post‐test evaluations commenced on the second day following its conclusion in order to standardize the process. There was no verbal encouragement given throughout the evaluations, and each participant finished all of the exams in a predetermined nonrandomized order with a 5‐min rest period in between. All participants mounted the horse using an access ramp with minimal assistance from staff.

With two sessions every week for 8 weeks, the intervention totaled sixteen hippotherapy sessions. This was a physical therapy intervention using hippotherapy (HPOT). In each session, participants rode a horse for 30 min. The first week's activities increased in complexity, starting with familiarization, during which participants adjusted to the horse's rhythmic movements while concentrating on lower limb and spine postural alignment. At this point, horses moved on flat grass in straight lines or in moderate circles.

**Figure 3:**
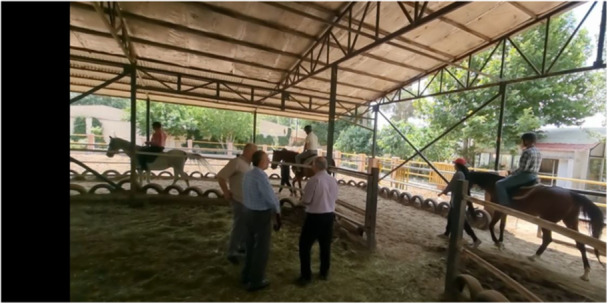
Training sessions during hippotherapy intervention.

From the second week, participants practiced different riding positions (front, left, right, inverted) and engaged in exercises for shoulder flexion and abduction. Difficulty increased gradually, culminating in riding in a saddle on sandy, sloped, and uneven terrain, incorporating trunk rotation. Stirrup adjustments ensured proper spinal and lower limb alignment. Exercises included bilateral knee extensions during sharp turns and serpentine patterns, often done with eyes closed. Each session concluded with 5 min of stretching over a standing horse, involving reaching towards the horse's neck, feet, and even touching the tail. The individuals would always begin and end the session on the access ramp for mount. Each individual used the same animal and guide during all training sessions. Sessions included a horse leader and two side walkers for safety, with a 1:1 therapist‐to‐rider ratio in individual settings. Four Horses (Educational) were used, selected for their calm temperament and even gait. Standard English saddles were employed. Not every participant rode a fixed horse. Moreover, the horses used in the hippotherapy were not scheduled in any other activity except their daily exercise, which consisted of walking around the area. In addition, the hippotherapy staff constantly observed the horses during the training sessions (Figure 3). The study was conducted in Tehran during the summer period, with average temperatures ranging from 20°C to 30°C and dry conditions. No adverse weather events or other incidents affected the activities or assessments. Participants missing > 10% of sessions were excluded; all remained compliant. During each hippotherapy session, the supervising physical therapist and the hippotherapy team systematically monitored participants for adverse events or unintended effects (e.g., falls, new or worsening musculoskeletal pain, dizziness, shortness of breath, or cardiovascular symptoms). Any event was to be documented in a standardized adverse‐event log and, where necessary, referred for medical evaluation. No harms were reported.

### 2.4 Statistical Analysis

The sample's age, weight, height, and body mass index (BMI) were characterized using the mean and standard deviation. Shapiro‐Wilk statistics (*p* > 0.05) and Levene's test (*p* > 0.05) were employed to verify the dependent variables' normal distribution and homogeneity. The General Linear Model of ANCOVA was used with a significance threshold of *p* = 0.05 to determine the impact of the various treatments on the functional variables (Assel et al., 2018). ANCOVA was chosen to adjust for pretest scores as covariates, increasing statistical power by controlling for baseline differences. All tests were two‐sided with α = 0.05. SPSS version 26.0 was used. We followed SAMPL guidelines for reporting. All outcome data were complete for all randomized participants; therefore, analyses were based on complete cases, and no imputation for missing data was required.

## 3 Results

The sample's description characteristics are shown in Table 1. At baseline, there were no discernible differences between the groups, and all dependent variables showed normalcy. Tables 2 and 3 present descriptive statistics and inferential analysis of the dependent variables, respectively. No adverse events or unintended effects related to the intervention were observed in either group.

The ANCOVA indicated that (I) Fall risk, (II) Stability index, (III) Balance index, (IV) Anterior posterior deviation, (V) Mediolateral deviation, presented a statistically significant Decrease compared to the control group, indicating an improvement in the balance.

After adjusting for pretest scores, there was a significant effect of the intervention on fall risk (F) = 42.87, *p* < 0.001, partial η² = 0.77, 95% CI [−5.84, −3.21]), stability index (F) = 2.22, *p* = 0.041, partial η² = 0.15, 95% CI [−1.62, −0.01]), anterior‐posterior sway (F) = 8.18, *p* = 0.013, partial η² = 0.39, 95% CI [−0.48, −0.07]), mediolateral sway (F) = 15.46, *p* = 0.002, partial η² = 0.54, 95% CI [−0.56, −0.15]), and balance index (F) = 9.63, *p* = 0.008, partial η² = 0.43, 95% CI [−1.02, −0.18]). These indicate improvements in the experimental group, with the largest effect on fall risk. The ANCOVA model confirmed all pre‐test variables as significant, demonstrating effective control of the covariate. Although other variables contributed to overall performance, the intervention was the primary catalyst for positive change. Additionally, the analysis showed that the interaction between the intervention and training groups significantly influenced outcomes, with training participants demonstrating improved performance.

## 4 Discussion

The purpose of this study was to examine how physical therapy with hippotherapy (HPOT) affected older people's balance. The investigation's main findings indicate that the intervention was successful in enhancing the participants' equilibrium, showing a noteworthy variation in each of the factors. Building on these statistical findings, the observed improvements in balance variables can be interpreted in the context of hippotherapy's neuromuscular stimulation.

In particular, we found that fall risk significantly improved as compared to other factors (Table 3). The motor changes brought about by the combination of the sensory stimulation from riding and hippotherapy can be responsible for the benefits of hippotherapy on balance in the elderly [19, 20]. Hippotherapy enhances balance through rhythmic equine movements stimulating proprioception and vestibular systems, improving variables like anterior‐posterior sway (important for fall prevention) [21, 22].

Furthermore, the data support the notion that engaging in hippotherapy not only enhances balance but also fosters greater confidence in mobility among elderly participants. The results highlight a marked improvement in both proprioceptive feedback and vestibular function, crucial aspects for maintaining stability. Moreover, individuals participating in the therapy report increased feelings of safety when navigating their environments. This correlation between therapeutic riding and enhanced risk perception suggests that hippotherapy could serve as a valuable adjunct to traditional physical rehabilitation programs for the elderly, ultimately contributing to a higher quality of life. Future research should focus on longitudinal studies to assess the sustained benefits of hippotherapy over time and explore its potential applications within diverse geriatric populations.

To our knowledge, there are only two studies on hippotherapy in elderly individuals, yielding different results than ours. Toigo, Junior, and A'vila (2008) examined the impact of eight hippotherapy sessions on the static balance of 10 elderly participants using a force platform. They found no significant difference in the acceleration speed of the center of pressure (COP) or in COP displacement in the medial–lateral direction, with improvements noted only in the anteroposterior COP [17]. However, due to the small, uncontrolled sample size, these results may not accurately reflect balance improvement. In a controlled trial by Araujo et al. [3], 8 weeks of hippotherapy also failed to significantly enhance stabilometric (relating to measurement of postural stability) variables (anteroposterior and mediolateral COP) in healthy elderly participants compared to the control group. Generally, the benefits of hippotherapy on balance have been observed primarily in patients with neurological disorders [3].

Kim et al. [23] studied the effects of 8 weeks of equestrian training on the balance of elderly men with Alzheimer's disease, finding significant improvements in both static and dynamic balance [23]. Kim and lee. (2015) reported notable enhancements in balance among elderly men post‐stroke following 8 weeks of equestrian training [24]. While our participants had no such diseases, these studies provide comparative evidence that hippotherapy's benefits may extend to healthy elderly, as seen in our results. Additionally, Kang and king (2015) explored the impact of horseback riding on the balance of elderly men with depression, while Raposo et al. [25] focused on those with knee osteoarthritis, both studies indicating significant improvements in balance indices [25, 26].

Systematic review investigations moreover affirm the positive impact of hippotherapy on balance and postural control in neurological patients. Zadnikar and Kastrin [27] illustrated the positive and critical impact in people with cerebral paralysis and Bronson et al. (2010) in people with different sclerosis [10, 27]. A significant Systematic review studies also confirm the positive effect of hippotherapy on balance and postural control in neurological patients. Zadnikar and Kastrin [27] demonstrated the positive and significant effect in individuals with cerebral palsy and Bronson et al. (2010) in individuals with multiple sclerosis [27, 28]. A significant improvement was documented in studies by Benda et al. [4], Bertoti [29], Kuczyn‐ski and Słonka [30], Quint and Toomey [31] and Shurtleff et al. [32], by MacKinnon et al. [33] in children with moderate CP, and by MacPhail et al. [34] in children with diplegic CP. Our study adds to this literature by demonstrating benefits in healthy elderly using dynamic Biodex measures.

Additionally, a study evaluated the static balance and gait of seniors, employing hippotherapy and treadmill training with 20‐min sessions conducted three times a week for 12 weeks. The researchers concluded that the elderly showed improved static balance following hippotherapy interventions [35]. The beneficial impact of hippotherapy on balance might stem from its dynamic nature, which includes oscillatory movements in lateral, vertical, and forward‐backward directions caused by the horse, leading to an enhancement in dynamic posture.

Hippotherapy, a therapeutic intervention involving horseback riding, has gained recognition for its efficacy in improving balance among individuals with various physical and neurological conditions. This methodology employs the rhythmic, three‐dimensional movement of the horse to stimulate the rider's sensory and motor pathways, thereby promoting improved balance and coordination [20]. Thus, hippotherapy improved several factors: fall risk, stability index, balance index, anterior posterior deviation, mediolateral deviation throughout the sessions.

The mechanism of hippotherapy's effect on balance can be elucidated through several key factors. Firstly, the horse's movement mimics the natural gait of a human, producing oscillatory forces that challenge the rider's postural control systems [21]. As the horse moves, it engages the rider's core and stabilizing muscles, facilitating neuromuscular re‐education necessary for maintaining equilibrium. This dynamic engagement enhances proprioception the body's ability to perceive its position in space thereby fostering better balance and coordination [22].

Secondly, hippotherapy encourages the activation of the vestibular system, which is crucial for spatial orientation and balance regulation. The interactions between the rider and the horse produce a unique sensory experience that stimulates vestibular inputs. Over time, this stimulation can lead to improved vestibular function, resulting in enhanced balance capabilities [36]. Furthermore, the psychosocial aspects of hippotherapy cannot be overlooked. The emotional bond formed with the horse can significantly boost confidence and motivation in individuals who might otherwise struggle with balance challenges. Increased self‐efficacy often translates to greater willingness to engage in physical activities, further reinforcing the balance improvements achieved through therapy [37].

In conclusion, the mechanisms underlying hippotherapy's positive effects on balance involve a complex interplay of physical, sensory, and psychological components. By harnessing the unique qualities of equine movement, hippotherapy provides a holistic approach to improving balance, ultimately enhancing the quality of life for many individuals. As research continues to expand in this field, it underscores the value of innovative therapeutic interventions in rehabilitation practices.

Our study has limitations, including a lack of control over certain health factors in elderly participants that could affect their responses to practical training, such as diet, medication use, and sleep duration. Additionally, we could not control the activities prior to assessment tests and training sessions. A follow‐up assessment was not conducted due to resource constraints; future studies should include 30‐day or longer follow‐ups to evaluate effect persistence. The study included only elderly males, limiting generalizability to females. Mounting assistance may limit applicability to those requiring more aid. These findings imply that hippotherapy could be integrated into geriatric rehabilitation programs, with future research exploring long‐term effects and diverse populations. This information is important for future research.

## 5 Conclusions

Current results indicate that physical therapy with hippotherapy (HPOT) significantly improves balance in physically active elderly individuals. This suggests that hippotherapy may enhance the quality of life for older adults by improving balance. The horse's three‐dimensional movement positively affects the human body, leading to better balance, which can further enhance gait and functional activity, thereby improving overall quality of life. Additional research on the long‐term effects of hippotherapy on aging is warranted.

## Author Contributions

**Bita Bahrami Gholami:** conceptualization, Investigation, methodology, validation, software, formal analysis, writing – original draft. **Ali Fatahi:** conceptualization, investigation, methodology, validation, visualization, writing – review and editing, resources, supervision, data curation, formal analysis, Software, Project administration. **Neda Boroushak:** conceptualization, project administration, methodology. **Rozhin Molavian:** writing – original draft, Software, formal analysis, project administration, data curation, supervision, resources.

## Conflicts of Interest

The authors declare no conflicts of interest.

## Transparency Statement

The lead author Ali Fatahi affirms that this manuscript is an honest, accurate, and transparent account of the study being reported; that no important aspects of the study have been omitted; and that any discrepancies from the study as planned (and, if relevant, registered) have been explained.

## Data Availability

The data presented in this study are available on request from the corresponding author.
